# Contribution of proteases to the hallmarks of aging and to age‐related neurodegeneration

**DOI:** 10.1111/acel.13603

**Published:** 2022-03-29

**Authors:** Mamta Rai, Michelle Curley, Zane Coleman, Fabio Demontis

**Affiliations:** ^1^ Department of Developmental Neurobiology St. Jude Children’s Research Hospital Memphis Tennessee USA

**Keywords:** aging, extracellular proteostasis, neurodegeneration, peptidase, protease, proteolysis

## Abstract

Protein quality control ensures the degradation of damaged and misfolded proteins. Derangement of proteostasis is a primary cause of aging and age‐associated diseases. The ubiquitin–proteasome and autophagy‐lysosome play key roles in proteostasis but, in addition to these systems, the human genome encodes for ~600 proteases, also known as peptidases. Here, we examine the role of proteases in aging and age‐related neurodegeneration. Proteases are present across cell compartments, including the extracellular space, and their substrates encompass cellular constituents, proteins with signaling functions, and misfolded proteins. Proteolytic processing by proteases can lead to changes in the activity and localization of substrates or to their degradation. Proteases cooperate with the autophagy‐lysosome and ubiquitin–proteasome systems but also have independent proteolytic roles that impact all hallmarks of cellular aging. Specifically, proteases regulate mitochondrial function, DNA damage repair, cellular senescence, nutrient sensing, stem cell properties and regeneration, protein quality control and stress responses, and intercellular signaling. The capacity of proteases to regulate cellular functions translates into important roles in preserving tissue homeostasis during aging. Consequently, proteases influence the onset and progression of age‐related pathologies and are important determinants of health span. Specifically, we examine how certain proteases promote the progression of Alzheimer's, Huntington's, and/or Parkinson's disease whereas other proteases protect from neurodegeneration. Mechanistically, cleavage by proteases can lead to the degradation of a pathogenic protein and hence impede disease pathogenesis. Alternatively, proteases can generate substrate byproducts with increased toxicity, which promote disease progression. Altogether, these studies indicate the importance of proteases in aging and age‐related neurodegeneration.

## Graphical abstract

Proteases are a large family of proteolytic enzymes located in all cell compartments and in the extracellular space. Proteases impact all hallmarks of cellular and tissue aging, and the onset and progression of age‐related diseases including neurodegeneration.

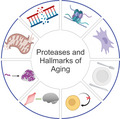

## 1 INTRODUCTION

**FIGURE 1:**
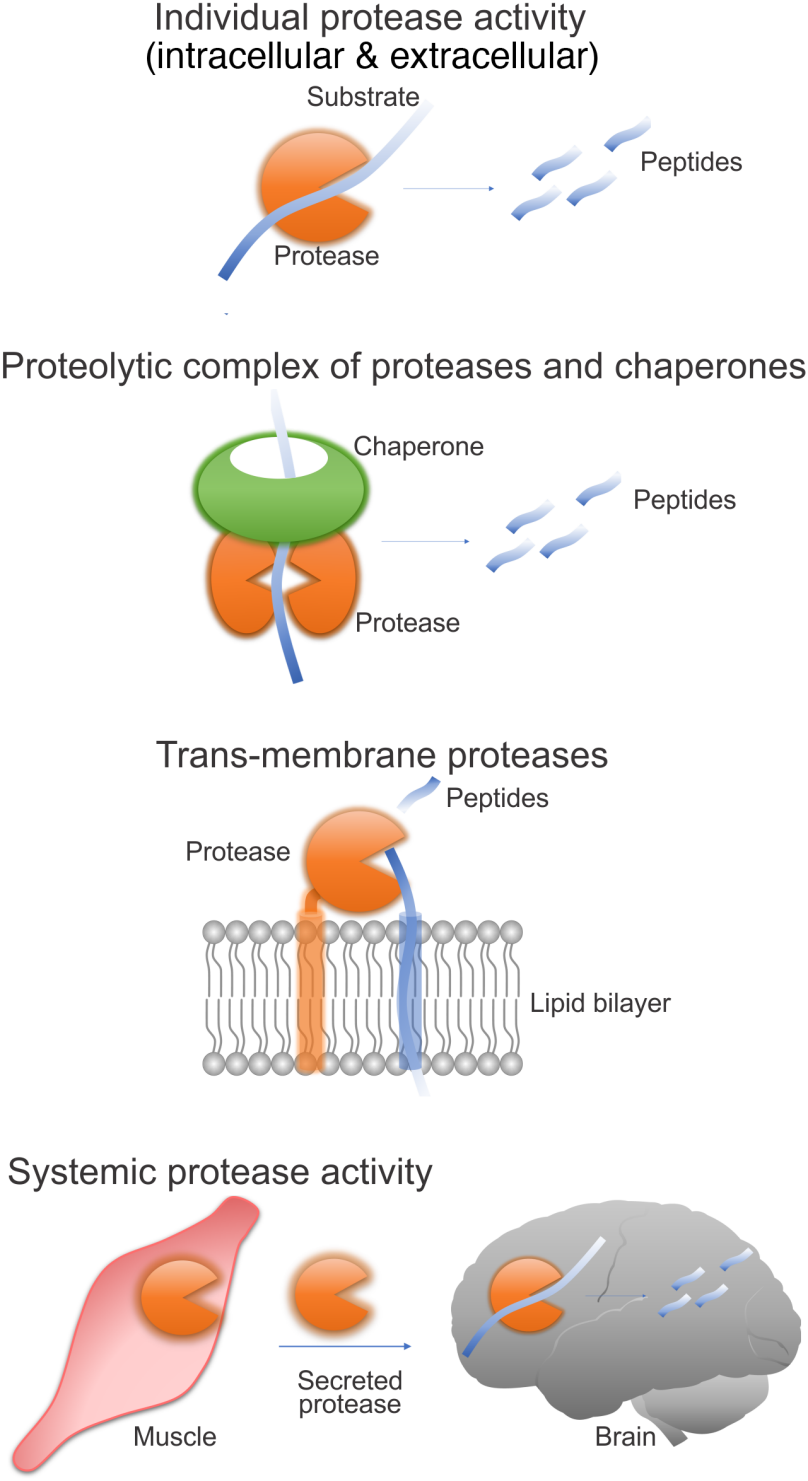
Modes of proteolysis by proteases. Proteases can enzymatically digest a substrate peptide/protein as a single enzyme or can form a proteolytic complex with chaperones. Proteases can as well be localized to cellular membranes to target membrane‐bound substrates or can be secreted to signal to distant organs

Protein degradation pathways have emerged as important modulators of aging via their capacity to degrade misfolded and aggregation‐prone proteins (Balch et al., 2008; Douglas & Dillin, 2010). Most of the studies that have established an important role of proteostasis in the aging process have focused on the role of the ubiquitin–proteasome and the autophagy‐lysosome systems. Interestingly, beyond these well‐established systems, there is a large class of proteolytic enzymes, that is, proteases/peptidases, with diverse intracellular and extracellular localization (Lopez‐Otin & Bond, 2008). These proteases can function as individual enzymes, hetero‐multimeric complexes with chaperones, transmembrane/intramembrane proteases, and/or can be secreted into the extracellular space (Figure 1).

**FIGURE 2:**
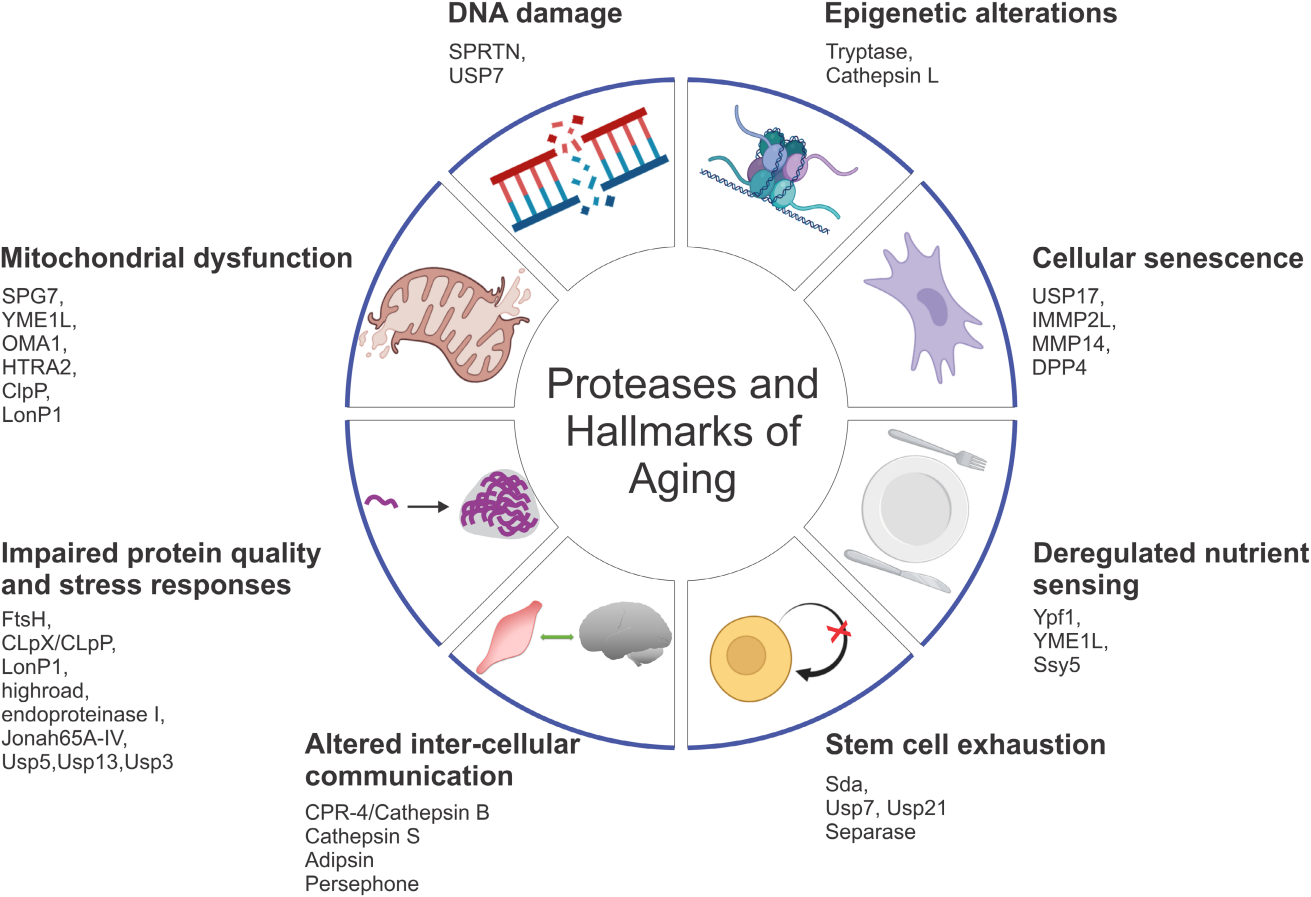
Proteases impact all hallmarks of aging. Altered protease activity can contribute to numerous hallmarks of aging, including mitochondrial dysfunction, DNA damage, epigenetic alterations, cellular senescence, deregulated nutrient sensing, stem cell exhaustion, altered intercellular communication, impaired protein quality control, and stress response. Some examples of these proteases are listed and discussed in the text. This scheme was drawn with BioRender

In addition to critical roles in cell function and survival, proteases directly contribute to the hallmarks of aging (Gottesman et al., 1997), which have been defined as genomic instability, telomere attrition, epigenetic alterations, loss of proteostasis, deregulated nutrient sensing, mitochondrial dysfunction, cellular senescence, altered intercellular communication, and stem cell exhaustion (Lopez‐Otin et al., 2013). Consequently, modulation of these proteolytic enzymes can influence the onset and progression of several age‐related pathologies, including neurodegeneration. In this review, we examine the role of proteases and provide examples of their contribution to several cellular processes relevant for aging and age‐related neurodegeneration (Figure 2).

## 2 OVERVIEW OF PROTEASES

Proteases can range from small enzymes made up of simple catalytic units (~20 kDa) to complex proteolytic machineries of 0.7–6 MDa that degrade target proteins by cleaving α‐peptide bonds and isopeptide bonds or that self‐hydrolyze their polypeptide chain (Lopez‐Otin & Bond, 2008).

Generally, proteases can be classified into two categories: endopeptidases and exopeptidases. However, with the advent of new structural and mechanistic information, a new classification now separates proteases into six different categories: aspartic, glutamic, cysteine, serine, threonine, and metalloproteases (Lopez‐Otin & Bond, 2008). Metalloproteases and serine proteases are the most abundant, with nearly 200 members each, as defined in MEROPS, a database for proteases and protease inhibitors. MEROPS currently describes 1129 human proteases, alongside their predicted cellular localization, although some of these entries represent pseudogenes derived from retroviral elements (Rawlings et al., 2008, 2016, 2018).

The Degradome database is another database that relies on exhaustive curation of individual entries for proteases/peptidases. Since the Degradome database does not include pseudogenes or retrovirus‐derived proteases, it lists only 588 human proteases (Lopez‐Otin & Bond, 2008). This database emphasizes on the pathological relevance of proteases to better understand disease (Quesada et al., 2009). There are also other databases, such as the ProLysED, which classify proteases based on different criteria (Quesada et al., 2009).

The availability of genome sequencing data from different animal and plant species has led to global surveys of the degradome and the comprehensive cross‐comparison of proteases across species. These analyses have shown that chimpanzees and humans have very similar degradomes, apart from differences in immune system proteases like caspase‐12 (Puente et al., 2005; Puente et al., 2005). Model organisms, such as rodents (Puente & Lopez‐Otin, 2004) and *Drosophila melanogaster*, have >600 proteases and many of these have orthologs in humans, suggesting that experimentally‐accessible model organisms could be useful to better understand fundamental functions of these proteases in aging.

This review aims at providing an overview of the role of proteases in key age‐associated processes that have been defined as hallmarks of aging. Because of the vastity of the protease family, which precludes an exhaustive account of all proteases involved in aging, this review aims at highlighting key examples of proteases that contribute to cellular and organismal aging (Figure 2 and Table 1), and how they impact neurodegeneration, an example of age‐related disease.

**TABLE 1:** Proteases that regulate hallmarks of aging

Section	Hallmarks of aging	Proteases involved
3.1	Mitochondrial dysfunction	YME1L, OMA1, HtrA2/Omi, CLpP/CLpX, LonP1, SPG7
3.2	DNA damage	Wss1/Spartan, USP7
3.3	Epigenetic alterations	Tryptase, Cathepsin L
3.4	Deregulated nutrient sensing	Ypf1/SPP, Ssy5, YME1L
3.5	Stem cell exhaustion	Usp21, Sda, LonP1, YME1L, Separase
3.6	Stress responses	TPPII, CG9733, CG7142, Usp5, Usp13, Usp3, Zmpste24
3.7	Impaired protein quality control	Endoproteinase I, Jonah65A‐IV, Highroad, Calpains, USP‐19, FtsH, ClpXP, LonP1
3.8	Altered intercellular communication	CPR‐4/Cathepsin B, Cathepsin S, Thrombin, Gingipains
3.9	Cellular senescence	USP17, IMMP2L, DPP4, MMP14, MMP‐1, MMP‐3, MMP‐10

## 3 CONTRIBUTION OF PROTEASES TO THE HALLMARKS OF AGING

### 3.1 Mitochondrial dysfunction

Mitochondrial dysfunction is one of the hallmarks of aging and is implicated in many age‐related diseases (Lopez‐Otin et al., 2013). A variety of cellular strategies exist to preserve mitochondrial function, including the selective degradation of non‐functional mitochondrial proteins via the ATPase Associated with diverse cellular Activities (AAA) family of mitochondrial proteases, which have been extensively studied in bacteria by virtue of their evolutionary conservation (Quiros et al., 2015). Mitochondrial proteases have been found to contribute to several human diseases (Gomez‐Fabra Gala & Vogtle, 2021) via their capacity to modulate mitochondrial protein trafficking, processing, and activation; mitochondrial protein quality control; the mitochondrial unfolded protein response; mitochondrial dynamics and mitophagy; and mitochondrial biogenesis (Deshwal et al., 2020; Quiros et al., 2015).

YME1L and OMA1 are mitochondrial inner membrane proteases that regulate mitochondrial morphology via proteolytic processing of the dynamin‐like GTPase OPA1. Processing of OPA1 by YME1L leads to the formation of the OPA1 *d* isoform, which promotes maintenance (or recovery) of tubular mitochondrial morphology. Conversely, OMA1‐mediated processing of OPA1 results in the production of the *c* and *e* isoforms, which promote mitochondrial fragmentation. Moreover, YME1L and OMA1 degrade each other in response to distinct types of toxic challenges: OMA1 is degraded by YME1L following insults that depolarize the mitochondrial membrane (but that do not deplete ATP); conversely, YME1L is degraded by OMA1 in response to insults that both depolarize the mitochondrial membrane and also deplete ATP. In this manner, these two proteases can differentially regulate mitochondrial dynamics in response to distinct types of stress (Rainbolt et al., 2016).

Another example of an important mitochondrial protease is HtrA2/Omi, which localizes to the intermembrane space. Its loss leads to accumulation of unfolded proteins in mitochondria, oxidative stress, and defective mitochondrial respiration. HtrA2/Omi insufficiency in mice is most often associated with early‐onset neurodegeneration and Parkinson's‐like phenotypes of *mnd2* (missense mutation) mice. Importantly, exogenous expression of human HtrA2 in the CNS of *mnd2* mice rescues neurodegeneration. However, insufficient HtrA2 activity in non‐CNS tissues results in premature aging phenotypes, that is, lordokyphosis, alopecia, weight loss, muscle atrophy, and induction of cell senescence (Kang et al., 2013).

In addition to proteases acting in the mitochondrial inner membrane and intermembrane space, there are also many examples of proteases that act in the mitochondrial matrix. Specifically, CLpP works along with the chaperone CLpX (caseinolytic protease subunit X) to degrade misfolded proteins in the mitochondrial matrix: CLpX recognizes protein substrates and unfolds and translocates them to CLpP for proteolysis. On this basis, CLpX/CLpP is induced by and is essential for resolving the mitochondrial unfolded protein response (UPR^mt^) in *Caenorhabditis elegans* and other organisms (Haynes et al., 2007; Kirstein‐Miles & Morimoto, 2010). Similar to CLpX/CLpP, other proteases such as LON (LONP1) display proteolytic activity and aid protein folding in close association with mtHSP70 in the mitochondrial matrix (Shin et al., 2021). Gene expression studies in the mouse have found that *LonP1* expression declines in the gastrocnemius muscle with aging (Lee et al., 1999) and that, conversely, *LonP1* expression is increased in the heart of mice selected for lifelong voluntary exercise (Bronikowski et al., 2003). Association of LonP1 to organismal health span has also been studied in the fungus *Podospora anserina* where constitutive overexpression of PaLON increases health span and resistance to oxidative stress (Luce & Osiewacz, 2009). In humans, pathogenic *LonP1* mutations are associated with cerebral, ocular, dental, auricular, skeletal (CODAS) syndrome resulting in multi‐organ malfunction, postnatal cataracts, skeletal deformities, hearing loss, and respiratory defects (Strauss et al., 2015). Lymphoblastoid cell lines generated from patients with *LonP1* mutations display altered mitochondrial morphology and reduced mitochondrial respiratory activity, leading to impaired mitochondrial proteostasis and function (Strauss et al., 2015). Additionally, knockout of *SPG7*, a metalloprotease that localizes to the mitochondrial matrix, shortens lifespan, exacerbates age‐related decline in neuromuscular function, and increases sensitivity to heat/mechanical/oxidative stress in *Drosophila* (Pareek et al., 2018).

Altogether, these studies exemplify the important functions of proteases located in the mitochondrial matrix, inner membrane, and intermembrane space in maintaining mitochondrial proteostasis and function during aging. Whereas proteases maintain proteostasis in inner mitochondrial compartments, the ubiquitin–proteasome system is responsible for the degradation of mitochondrial outer membrane proteins (den Brave et al., 2021). However, it was recently suggested that mitochondrial proteases may aid the ubiquitin/proteasome system in maintaining proteostasis also by degrading misfolded proteins that originate from other cell compartments (Ruan et al., 2017). In this scenario, the disaggregase Hsp104 dissociates proteins from aggregates to facilitate their entry into mitochondria, followed by degradation of such aggregation‐prone proteins by mitochondrial matrix proteases including LON (Ruan et al., 2017).

Therefore, this study highlights that there are unexpected ways via which mitochondrial proteases regulate cellular proteostasis. Moreover, despite extensive knowledge about mitochondrial proteases, it is only partially known how their activity is controlled and how it could be potentiated to contrast mitochondrial dysfunction with aging.

### 3.2 DNA damage

Beyond mitochondria, the nucleus is another important cellular compartment in which proteostasis is modulated by proteases, in addition to the ubiquitin–proteasome system. DNA damage is a major hallmark of aging and several nuclear proteases have been found to regulate it, as explained here below.

DNA‐protein crosslinks (DPCs) are highly toxic DNA adducts that can cause cellular and organismal stress by hindering proper DNA replication. If not repaired, DPCs can impair genomic stability, which leads to premature aging syndromes and cancer (Ruggiano & Ramadan, 2021; Stingele & Jentsch, 2015). An important study has found a key role for the yeast protease Wss1 and its higher‐organism ortholog Spartan (SPRTN) in DPC repair. Specifically, Wss1 cleaves the proteinaceous part of DPCs in a DNA‐dependent manner to clear covalently bonded TOP1 adducts (Stingele et al., 2014). A subsequent study further demonstrated the relative roles of SPRTN versus the proteasome in DPC repair in *Xenopus* egg extracts. It was found that SPRTN and the proteasome are distinct pathways that act independently from each other (Larsen et al., 2019; Ruggiano et al., 2021). SPRTN recruitment requires DPC SUMOylation (Ruggiano et al., 2021) and the presence of a DNA polymerase on one side and a short tract of ssDNA on the other side of a DPC in order to prevent indiscriminate destruction of replisome components and chromatin proteins (Larsen et al., 2019).

Another set of studies has found a role for SPRTN in the activation of the DNA damage stress response. Specifically, SPRTN activates the ATR‐CHK1 phosphorylation signaling cascade by cleaving the inhibitory C‐terminal part of the CHK1 kinase, which in turn phosphorylates SPRTN and promotes its recruitment to chromatin to promote DPC repair (Halder et al., 2019). In another study, SPRTN was found to interact with the ubiquitinated proliferating cell nuclear antigen (PCNA), which increases upon UV irradiation and DNA damage and serves as a molecular mark to guide post‐replication repair (Centore et al., 2012). Interestingly, the interaction of SPRTN with PCNA was needed to increase PCNA ubiquitination by the E3 ubiquitin ligase Rad18 and hence reinforce the UV‐induced DNA damage response, apparently in a manner independent from the proteolytic function of SPRTN (Centore et al., 2012).

Consistent with these studies, *Sprtn* ^−/−^ mouse embryonic fibroblasts exhibit cell cycle arrest prior to mitosis, DDR activation, genomic instability (i.e., chromatin bridges and micronuclei), and decreased stress resistance. Although Spartan knockout mice are early embryonic lethal, hypomorphic mutants are viable and display genomic instability and premature aging phenotypes typical of progeroid syndromes, that is, lordokyphosis, cataracts, body wasting (cachexia), accumulation of senescent cells in the white adipose tissue, and decreased exercise capacity (Maskey et al., 2014). Consistently, germline mutations in *SPRTN* were identified in patients with a progeroid syndrome characterized by genomic instability and susceptibility toward early‐onset hepatocellular carcinoma (Ruijs‐Aalfs Syndrome/RJALS). Transfection of cultured patient cells with wild‐type SPRTN almost completely corrected replication defects and restored cellular proliferation in culture (Lessel et al., 2014).

Beyond proteases that act on DNA‐protein crosslinks, other proteases that maintain genome integrity act in the double‐strand break (DSB) repair pathways of homologous recombination (HR) and non‐homologous end joining (NHEJ). Maintaining a balance between these two pathways and ensuring that neither is overactive is critically important within a cell: Excessive NHEJ can result in mutagenesis whereas unrestrained HR can be cytotoxic. Ubiquitin proteases (DUBs) have important roles in fine‐tuning these processes, for example, in regulating turnover, activity, and protein–protein interactions of DSB repair factors, and this ensures efficient DSB repair and balance between HR and NHEJ pathways (Garvin, 2019).

An example of DUB that modulates DNA repair is USP7. The MRE11‐RAD50‐NBS1 (MRN) complex and mediator of DNA damage checkpoint protein 1 (MDC1) are essential for the repair of DSBs as they mediate the initiation and amplification of the DNA damage response (DDR). This study shows that the ubiquitin‐specific protease USP7 directly interacts with MRN‐MDC1. This interaction results in the de‐ubiquitination of MDC1 by USP7, thereby increasing the half‐life of MDC1. This study also found that upregulation of USP7 in cervical cancer protects cells from genotoxic insults by sustaining the DDR via MDC1 stabilization (Su et al., 2018). Altogether, these studies indicate the important roles of proteases in the DNA damage response and in preserving genome integrity.

### 3.3 Epigenetic alterations

Post‐translational histone modifications, histone exchange, and ATP‐dependent chromatin remodeling are key determinants of gene expression. Age‐dependent alteration of chromatin and DNA accessibility result in gene expression changes and compromise genomic stability (Pal & Tyler, 2016). In this context, the cleavage of histone tails by proteases is emerging as a novel mechanism of epigenetic regulation of gene expression during aging and disease (Yi & Kim, 2018).

An example of such regulation comes from allergy‐associated mast cell activation, which leads to the release of many signaling factors, including mast cell‐specific proteases such as tryptase. Interestingly, tryptase also localizes to nuclei of mast cells where it truncates H3 and H2B histones at their N‐termini (Melo et al., 2017). Mast cells (MCs) derived from the bone marrow of tryptase‐null mice display an age‐related increase in the levels of lysine 5‐acetylated H2B. As a result, tryptase‐deficient cells show profound upregulation of markers of non‐mast cell lineages, deranged chromatin remodeling, and consequent extensive morphological alterations. Additionally, tryptase‐null cells exhibit age‐dependent loss of proliferative control, which leads to a hyperproliferative state compared to MCs obtained from wild‐type mice. Reducing tryptase levels in wild‐type cells mimics the epigenetic changes that occur in tryptase‐null cells with aging, indicating that histone clipping by tryptase is a crucial age‐dependent determinant of epigenetic regulation (Melo et al., 2017).

N‐terminal histone tail cleavage by proteases has also been implicated also in cell senescence. Specifically, cathepsin L cleaves histone H3.3 variants at 2 sites and generates H3.3cs1, a major cleavage product that lacks the first 21 amino acids of the H3 tail (Duarte et al., 2014). Both mRNA and protein levels of cathepsin L increase upon senescence in human fibroblasts and melanocytes. Chemical and siRNA‐based inhibition of cathepsin L and subsequent inhibition of H3 cleavage impairs senescent‐associated chromatin modifications. Conversely, ectopic expression of H3.3cs1 is sufficient to induce senescence via transcriptional silencing of cell cycle regulators such as RB/E2F genes, likely via permanent removal of H3K4me3 (Duarte et al., 2014). Overall, these studies indicate that age‐dependent proteolytic cleavage of histone tails by proteases is a key regulator of epigenetic changes associated with aging and with cell senescence.

### 3.4 Deregulated nutrient sensing

Lysosomal proteases are key for the catabolic functions of lysosomes and the consequent release of lysosomal metabolites, which participate in nutrient sensing (Carmona‐Gutierrez et al., 2016). In addition, there are non‐lysosomal proteases involved in nutrient sensing (Chantranupong et al., 2015). These include Ypf1/SPP, an ER‐resident aspartyl intramembrane protease with a presenilin fold. In yeast, it was found that Ypf1 regulates the abundance of transmembrane nutrient transporters (Avci et al., 2014). At high zinc levels, the E3 ubiquitin ligase Doa10 ubiquitinates the cytosolic loop 3 of the zinc transporter Zrt1 and the protease Ypf1 drives the ubiquitin‐mediated degradation of Zrt1 by cleaving it and destabilizing its folding (Avci et al., 2014). Such proteolytic activity depends on the interaction of Ypf1 with Doa10 and with the ER‐associated degradation (ERAD) factor Dfm1. Altogether, it was proposed that Ypf1 contributes to a regulatory ERAD that adjusts the levels of nutrient transporters to cope with nutrient abundance (Avci et al., 2014). Other intramembrane proteases have been found to clip transmembrane proteins and promote their subsequent efficient degradation by the proteasome (Fleig et al., 2012; Greenblatt et al., 2012), suggesting that this might be a widespread mechanism to respond to environmental and nutrient challenges.

In addition to modulating the levels of nutrient transporters, proteases have been found to modulate also nutrient‐induced adaptive signaling. In yeast, the Ssy1 plasma membrane amino acid sensor detects the external amino acid concentration and transmits intracellular signals that modulate adaptive gene expression. A key component of this nutrient‐sensing system is the protease Ssy5, which changes conformation in response to the binding of Ssy1 to amino acids. Consequently, this leads to degradation of the Ssy5 pro‐domain and its activation. In turn, Ssy5 proteolytically cleaves and activates the transcription factors Stp1 and Stp2, which translocate to the nucleus and regulate the expression of amino acid permease genes (Abdel‐Sater et al., 2004, 2011; Andreasson et al., 2006; Omnus et al., 2011).

Beyond nutrient sensing, proteases have also been found to globally adapt the proteome to the energy requirements of the cell. In this scenario, the mitochondrial protease YME1L reshapes the mitochondrial proteome following nutrient starvation. Mitochondrial proteolysis is initiated by decreased mTORC1 activity and modulation of the phosphatidic acid phosphatase LIPIN1, which decreases phosphatidylethanolamine levels in mitochondrial membranes. Such change in the lipid composition of mitochondrial membranes promotes the proteolytic activity of YME1L, which degrades mitochondrial transporters and metabolic enzymes to reduce mitochondrial biogenesis and spare resources to support cell growth (MacVicar et al., 2019).

Altogether, these studies exemplify the role of proteases in nutrient sensing. However, it is currently unexplored whereas the altered function of these proteases is responsible for derangement of nutrient sensing during aging and age‐related metabolic disease or, conversely, they are required for anti‐aging interventions such as dietary restriction.

### 3.5 Stem cell exhaustion

Reduced regenerative capacity and stem cell dysfunction are a hallmark of aging and there are several examples of proteases that regulate stem cell function. These include ubiquitin proteases (i.e., deubiquitinating enzymes, DUBs), which also regulate other hallmarks of aging, as discussed above. The DUB Usp21 maintains the pluripotency of embryonic stem cells by increasing the levels of NANOG, a homeobox transcription factor that governs their self‐renewal and pluripotency. This occurs via removal of ubiquitin from NANOG by Usp21 and consequent reduction in NANOG proteasomal degradation (Kwon et al., 2017).

Another example of protease involved in stem cell function was found in the *Drosophila* testicular niche. In this system, the aminopeptidase Slamdance (Sda) maintain germline stem cells (GSCs) by ensuring the dedifferentiation of progenitor germ cells. Loss of Sda leads to niche deterioration and stem cell exhaustion, primarily due to the capacity of Sda to promote the accumulation of mature E‐cadherin. Consistently, Sda overexpression promotes dedifferentiation during aging and maintenance of GSCs (Lim et al., 2015).

Additional evidence for proteases in the maintenance of germline stem cells was also found in mice. By examining oocytes from conditional *Lonp1*‐knockout mice, it was found that the mitochondrial protease Lonp is necessary for oocyte survival: Lonp loss leads to progressive oocyte death, ovarian reserve decline, and infertility. Normally, Lonp1 inactivates Aifm1 (apoptosis‐inducing factor mitochondria associated 1), presumably via degradation. However, Lonp1 loss leads to Aifm1‐mediated apoptosis of oocytes. Similarly, pathogenic *LONP1* variants lead to infertility and premature ovarian insufficiency in women (Sheng et al., 2022).

Another mitochondrial protease, YME1L, regulates the quiescence and activation of neural stem and progenitor cells (NSPCs). YME1L preserves NSPC self‐renewal by regulating the abundance of numerous mitochondrial proteins, as explained in the paragraph above. YME1L deletion reduces fatty acid β‐oxidation, induces premature NSPC differentiation, and this eventually leads to NSPC pool depletion (Wani et al., 2022).

A role for proteases in stem cell aging has also been inferred from the emergence of age‐associated comorbidities in AIDS patients treated with protease inhibitors. In fact, protease inhibitors used for the treatment of HIV infections have been found to induce deleterious side effects, many of which are typically age‐associated conditions (such as osteoporosis). Mechanistically, protease inhibitors induced senescence and premature aging of endothelial cells, human bone marrow mesenchymal stem cells, and of osteoblast precursors (Hernandez‐Vallejo et al., 2013). Overall, these studies exemplify multiple mechanisms by which proteases regulate stem cell properties and regenerative capacity.

### 3.6 Role of proteases in stress responses

Several studies have found a role for proteases in stress responses. The proteasome is a major proteolytic system. When proteasome function is challenged, a condition known as proteasome stress, an adaptive stress response is induced which consists in the expression of non‐proteasomal proteases, which partially compensate for proteasome dysfunction via their capacity to degrade polypeptides by exo‐ and endo‐proteolytic cleavage (Geier et al., 1999; Glas et al., 1998; Lipinszki et al., 2013; Wang et al., 2000), as explained in detail here below.

Chemical inhibition of proteasome function is a stress for eukaryotic cells. However, a minority of cells recover and adapt to reduced proteasome function. Such stress‐adapted cells exhibit higher alanyl‐alanyl‐phenylalanyl‐ (or AAF‐) hydrolyzing activity and inhibiting this activity hinders the growth of the adapted cells but not of control cells (Glas et al., 1998). Tripeptidyl peptidase (TPPII) was identified as a stress‐responsive protease with AAF‐hydrolyzing activity that increases in cells resistant to proteasome inhibitors (Geier et al., 1999). TPPII is a large serine protease of the subtilisin‐type that degrades oligopeptides by both exo‐ and endopeptidase activity (Geier et al., 1999). Consequently, TPPII overexpression prevents the accumulation of polyubiquitinated proteins in cells exposed to a lethal dose of proteasome inhibitors and preserves normal cellular function and viability (Wang et al., 2000).

**FIGURE 3:**
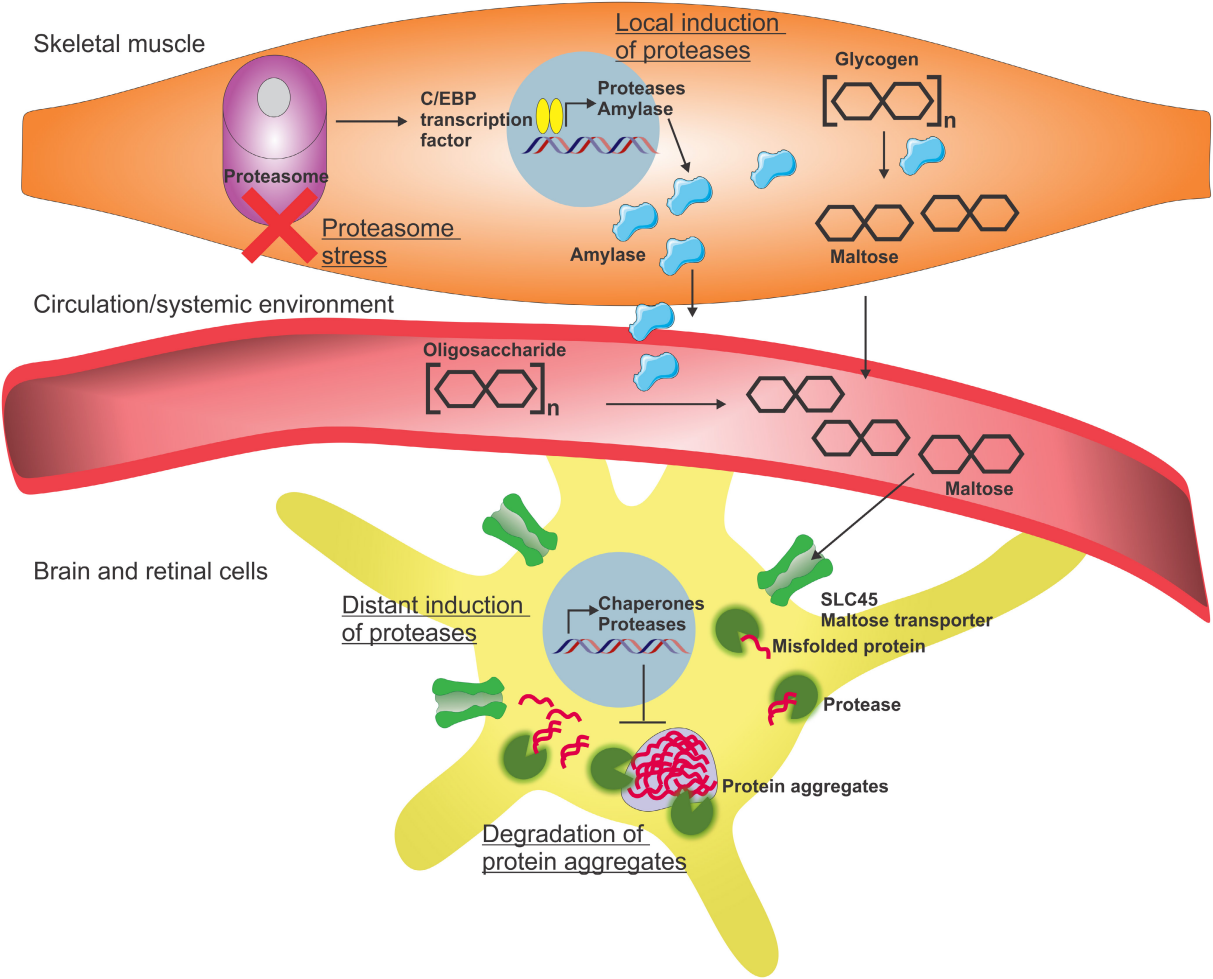
Proteases contribute to systemic adaptive stress signaling induced by proteasome stress in skeletal muscle. Knock‐down of proteasome subunits in skeletal muscles leads to a compensatory C/EBP‐dependent transcriptional program in the skeletal muscles and in the CNS (brain and retina). In skeletal muscle, proteasome stress induces expression of proteases and of a secreted amylase, Amyrel, which acts in the circulation and possibly also within skeletal muscle. Amyrel metabolizes poly/oligosaccharides into maltose and increases maltose systemic levels. Intracellular transport of maltose into CNS cells via SLC45 transporters induces the expression of proteases and chaperones that prevent age‐dependent increase in protein aggregates

Similarly, it was found in *Drosophila* that experimental induction of proteasome stress in skeletal muscle (via muscle‐specific RNAi for proteasome components) leads to local and systemic adaptations that include the expression of proteases in skeletal muscle and systemically in the central nervous system (Rai et al., 2021). Whereas the local induction of proteases in skeletal muscle likely depends on stress‐activated C/EBP transcription factors (Rai et al., 2021), induction of proteases in distant tissues relies on the muscle‐secreted amylase Amyrel, which promotes the expression of the *CG9733* and *CG7142* proteases. Importantly, RNAi‐mediated knockdown of CG9733 and CG7142 in the retina increases the amount of aggregates of pathogenic Huntingtin‐72Q during aging, suggesting that these proteases indeed preserve proteostasis in response to adaptive stress signaling mediated by Amyrel (Rai et al., 2021). Altogether, this study highlights stress‐induced adaptive signaling that preserves proteostasis during aging in skeletal muscle as well as in the retina and brain and that at least in part depends on proteases (Figure 3).

There are also several proteases that have been implicated in ubiquitin‐dependent stress granule assembly, that is, the transient aggregates of RNA‐binding proteins that rapidly form in response to a variety of cellular stressors. Specifically, USP5 exhibits proteolytic activity on free ubiquitin in human cells whereas USP13 targets ubiquitin attached to protein substrates: Together, they regulate heat‐induced stress granules by hydrolyzing ubiquitin chains (Xie et al., 2018). Likewise, Usp3 is another ubiquitin protease required for heat‐induced stress granule assembly in yeast cells (Nostramo et al., 2016).

Another example of stress response linked with proteases comes from studies on the zinc metalloproteinase Zmpste24 (the mouse ortholog of human FACE‐1). Different from the examples above, the protease Zmpste24 does not mediate a stress response, rather one is induced upon its loss. Normally, Zmpste24 is involved in the maturation of Lamin A (Lmna), which is an essential component of the nuclear envelope. Loss of Zmpste24 impedes the final cleavage step of prelamin A, resulting in a partially processed farnesyl full‐length prelamin A instead of mature Lamin A (Navarro et al., 2004, 2005; Spear et al., 2018). Similarly, a severe progeroid syndrome, restrictive dermopathy (RD), is caused by Zmpste24 deficiency in humans (Navarro et al., 2004, 2005; Spear et al., 2018). Consistent with the fact that they work in the same pathway, Zmpste24‐ and Lmna‐deficient mice exhibit a similar phenotype, that is, profound nuclear architecture abnormalities. Zmpste24 deficiency is marked by upregulation of p53 that leads to cellular senescence and accelerated organismal aging. Importantly, p53 loss partially reverses the phenotype of Zmpste24 null mice (Pendas et al., 2002; Varela et al., 2005), indicating that the stress response induced by Zmpste24 loss is maladaptive and contributes to accelerated aging.

### 3.7 Additional roles of proteases in protein quality control

Proteases contribute to the hallmarks of aging listed above primarily via their proteolytic action. In some cases, such proteolysis is regulatory, that is, leads to changes in the activity or in the levels of protein substrates, which modulates signal transduction. In other cases, proteases target misfolded and aggregation‐prone proteins, and hence, their action maintains protein quality control. Here below, we review additional examples of the role of proteases in proteostasis.

Analysis of *Drosophila* development has led to the identification of proteases that are relevant for maintaining protein quality control during growth. Specifically, endoproteinase I is a protease that is highly expressed in the early stages of larval development. During this stage, there is extensive growth of larval tissues and the whole body. The conclusion of larval development coincides with a sharp decline in endoproteinase I expression. Because endoproteinase I targets the proteasomal component Rpn10, this protease may regulate the function of the proteasome during developmental growth. Consistent with important roles for endoproteinase I, its knockdown results in pupal lethality. Another larval protease, Jonah65A‐IV, degrades only unfolded proteins and exhibits similar crosstalk with the proteasome. Altogether, these findings indicate that these proteases may be needed to maintain protein quality control in anabolic conditions, such as during the extensive tissue and body growth that occurs during the larval stages of *Drosophila* development (Lipinszki et al., 2013).

Proteases have been found to maintain protein quality control also in photoreceptors, where proteostasis is challenged by mutant disease‐associated proteins and by light exposure. A study in *Drosophila* photoreceptors has found that the protease *highroad* is required for the degradation of misfolding‐prone mutant Rhodopsin‐1. Ablation of the *highroad* protease accelerates age‐related retinal degeneration due to Rhodopsin‐1 mutants. Interestingly, it seems that *highroad* is part of an adaptive stress response that is induced to preserve proteostasis when challenged by mutant Rhodopsin: *Highroad* expression is induced by Rhodopsin mutants and requires dietary retinoids, indicating that the transcriptional induction of this protective protease is responsive to both stress and nutrient cues (Huang et al., 2018).

Proteolysis is important to maintain protein quality control and hence impede the accumulation of misfolded proteins that derange cellular function. However, in certain conditions, bulk non‐selective proteolysis can be deleterious. An example is the indiscriminate loss of muscle mass that occurs during atrophy induced by many diseases (Bonaldo & Sandri, 2013; Piccirillo et al., 2014). While it was recently found that preserving proteostasis is important for combating age‐associated atrophy, that is, sarcopenia (Demontis et al., 2013; Hunt et al., 2021; Jiao & Demontis, 2017), indiscriminate proteolysis has been reported to be deleterious. Specifically, calpains are calcium‐dependent proteases that have been found to promote skeletal muscle atrophy with aging, unloading, and sepsis. This is believed to occur because calpains can degrade bulk muscle constituents such as myofibrillar and cytoskeletal proteins. Consistently, overexpressing calpastatin, an endogenous calpain inhibitor, ameliorates muscle weakness associated with aging and also increases longevity (Schroder et al., 2021).

Muscle weakness also occurs as a result of motor neuron degeneration in amyotrophic lateral sclerosis (ALS), spinal muscular atrophy (SMA), and Charcot Marie Tooth type 2A (CMT2A). Mitofusin 2 (Mfn2), a protein required for outer mitochondrial membrane fusion, is implicated in CMT2A as it regulates the levels of calpastatin through mitochondrial trafficking‐dependent axonal transport. Importantly, genetic ablation of calpastatin in neurons obliterates Mfn2‐mediated protection of neuromuscular junctions (NMJs) in skeletal muscles and leads to age‐dependent muscle atrophy in mice (Wang et al., 2018). Similarly, higher calpain activity is observed in diseased motoneurons of in vitro models of SMA, and its inhibition is protective (de la Fuente et al., 2020).

Another example is the ubiquitin‐specific protease USP19, a deubiquitinating enzyme. USP19 expression correlates with atrophy markers such as MuRF1 and MAFbx/atrogin‐1 in muscle biopsies of cancer patients, suggesting participation of USP19 to atrophy‐associated protein degradation (Bedard et al., 2015). Indeed, USP19 knockout mice retain both muscle mass and strength and are protected against glucocorticoid‐ and denervation‐induced muscle atrophy (Bedard et al., 2015). Altogether, these studies indicate that unselective proteolysis mediated by proteases and directed toward bulk cellular constituents is generally deleterious and may contribute to muscle atrophy during aging.

Although studies in multicellular organisms have provided important insight into the role of proteases in tissue aging and age‐related disease, important insight into the function of proteases in disease‐relevant processes has been gathered also from studies in unicellular organisms such as yeast and bacteria. An example of such studies comes from the opportunistic bacterial pathogen *Pseudomonas aeruginosa*, for which protein degradation plays a major role in survival (Basta et al., 2020). When nutrients in their environment are exhausted, bacterial growth ceases because of nutrient scarcity. In this context, the heat‐shock protease FtsH is required for growth arrest survival of *P. aeruginosa*. Like FtsH, loss of other proteases better known for their roles in proteostasis in response to heat shock (“heat‐shock proteases”) induces lethality during growth arrest. Individual deletions revealed that FtsH and ClpXP proteases have primary, nonredundant roles, whereas HslVU and Lon are only secondarily required. These findings highlight potentially conserved functions for proteases in preserving proteostasis from bacteria to humans (Basta et al., 2020).

### 3.8 Altered intercellular communication

**FIGURE 4:**
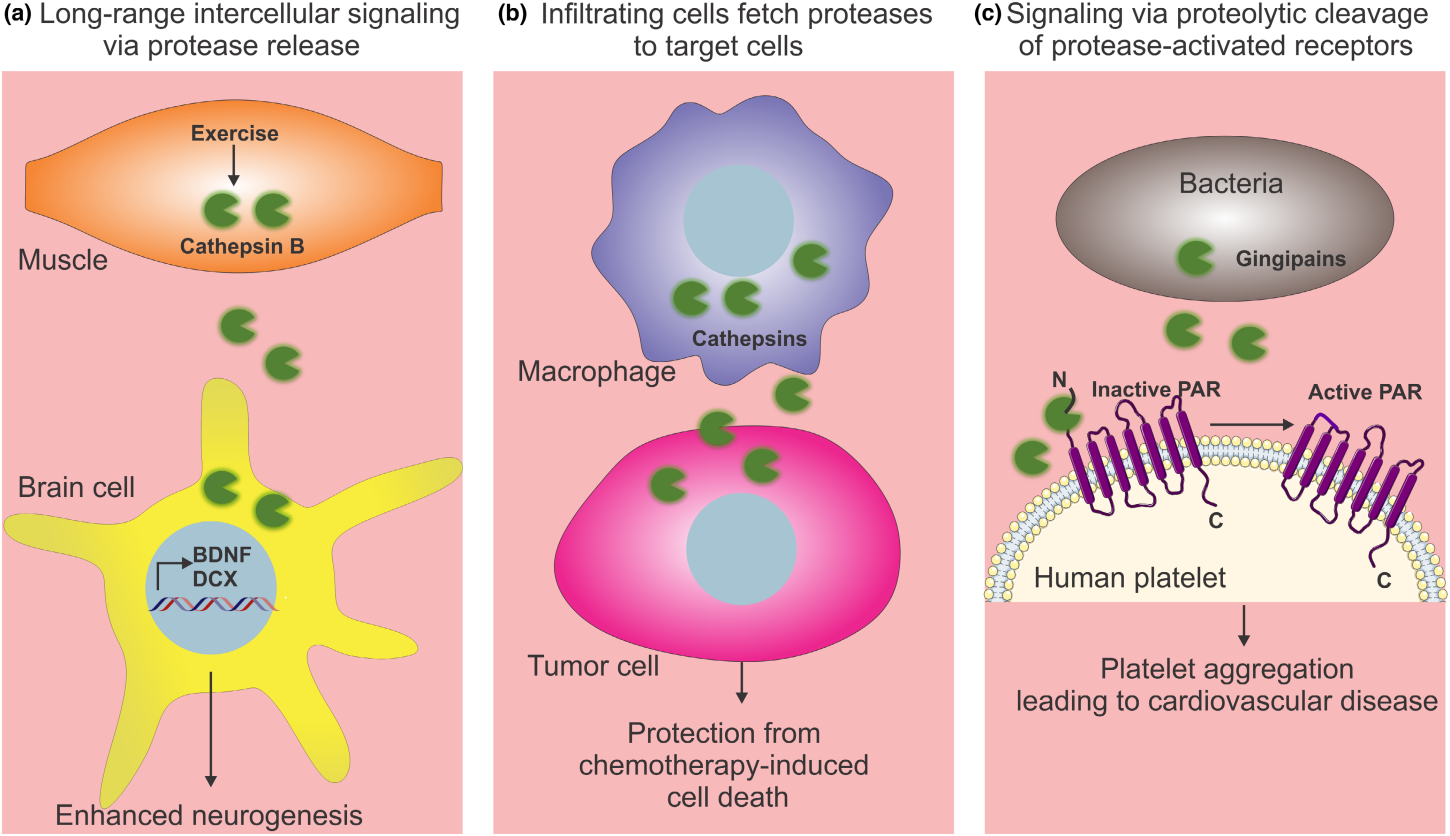
Role of proteases in intercellular signaling. There are three modes of intercellular signaling by proteases: (a) Long‐range signaling between distant tissues via secretory proteases. For example, skeletal muscle secretes cathepsin B in response to exercise, which in turn regulates brain neurogenesis and memory. (b) Inflammatory cells that infiltrate injured tissues and tumor cells secrete proteases that can be uptaken by target cells. (c) Secreted proteases can also proteolytically cleave protease‐activated receptors on target cells, leading to activation of the receptor and subsequent signal transduction

An important component of aging is the derangement of systemic signaling. Because proteases are located in all cellular compartments and in the extracellular space, they can influence extracellular proteolysis and hence inter‐tissue signaling. Additionally, apart from proteolysis, proteases can also function in an endocrine manner by acting as signaling molecules that regulate homeostasis during aging and disease (Figure 4).

An example of such a role comes from *C*. *elegans* whereby UV irradiation initiates an organismal stress response termed radiation‐induced bystander effect (RIBE) which participates in stress signaling initiated in the irradiated tissue and exerts its effects on distal tissues not exposed to UV. The cysteine protease CPR‐4, homologous to human cathepsin B, was found to be secreted extracellularly upon UV or gamma irradiation (Peng et al., 2017). Wild‐type worms treated with culture medium from irradiated animals show enhanced lethality, indicative of RIBE. Conversely, media from cpr‐4 RNAi‐treated animals reduces RIBE, an effect that is reversed by a cpr‐4 transgene. Mutations in the catalytic region of CPR‐4 inhibit both its protease activity and RIBE. In order to assess the relay of stress responses to distal organs, a radiation‐responsive reporter, Phsp‐4::gfp, was used. Localized UV irradiation in the head of wild‐type animals led to strong activation of the stress‐responsive reporter in the posterior part of the animal, inhibition of germ cell death in posterior gonads, and embryonic lethality in unirradiated embryos (Peng et al., 2017). Additionally, mCherry‐tagged CPR‐4 expressed specifically by pharynx muscles was secreted extracellularly and taken up by distant cells and, as observed in irradiation experiments, this resulted in decreased germ cell death and increased embryonic lethality. This systemic stress response was lost upon cpr‐4 RNAi, confirming that the CPR‐4 mediates a stress‐induced adaptive response (Peng et al., 2017). Inactivation of DAF‐2, the homolog of the insulin/IGF receptor, and PDK‐1 kinase, prevents long‐range stress signaling although the CPR‐4 secretion is not affected, suggesting that CPR‐4 exerts RIBE activity through DAF‐2 (Peng et al., 2017).

Interestingly, the human homolog of CPR‐4, cathepsin B (CTSB), was also identified as an exercise‐induced myokine in an in vitro exercise model (Moon et al., 2016). Running increases CTSB expression and protein levels both in the plasma and gastrocnemius muscles of mice. Furthermore, recombinant CTSB treatment stimulates the expression of brain‐derived neurotrophic factor (BDNF) and doublecortin (DCX) in adult hippocampal progenitor cells (Figure 4a). Running is known to induce adult neurogenesis in the dentate gyrus of the hippocampus and is positively associated with memory, but *ctsb* ^−/−^ knockout mice lack these benefits deriving from running. Similarly, treadmill exercise in monkeys and humans increases plasma CTSB levels and correlates with exercise benefits of fitness and hippocampal memory (Moon et al., 2016; Rai & Demontis, 2022). However, it is presently unclear whether these effects rely on the proteolytic activity of cathepsin B or whether cathepsin B acts as a signaling factor.

Although cathepsins are known for their intracellular (lysosomal) proteolytic actions, other studies have also hinted at possible extracellular roles of cathepsins. Specifically, extracellular cathepsins have been found to inhibit chemotherapy‐induced apoptosis in human breast cancers (Shree et al., 2011). Indeed, co‐culturing experiments revealed that macrophage‐secreted cathepsin protects tumor cells against chemotherapy‐induced cell death, and that this effect is prevented by cathepsin inhibitors. Macrophages from mice deficient for cathepsin B and S are impaired in their ability to prevent cell death in tumor cells upon taxol treatment, compared to the wild‐type macrophages, indicating that cathepsins B and S are key proteases necessary for this process (Figure 4b). Importantly, combinatorial chemotherapy with cathepsin inhibitors significantly enhances treatment of primary and metastatic tumors as well as improves late‐stage survival in mouse models (Shree et al., 2011).

Although it remains currently undetermined whether the proteolytic activity of cathepsin B is required for its signaling functions, there is evidence for protease‐mediated modulation of signal transduction. Specifically, proteases secreted in the extracellular space can activate protease‐activated receptors (PARs) by cleaving their extracellular domain on a target cell (Mackie et al., 2002). The four known PARs belong to the family of seven‐transmembrane domain G protein‐coupled receptors and activate intracellular signaling pathways typical for this family of receptors. PAR‐1, PAR‐3, and PAR‐4 are mostly activated by thrombin whereas PAR‐2 is activated by a variety of proteases, including trypsin and tryptase (Mackie et al., 2002). Thrombin is a serine protease that is generated at sites of vascular injury and acts on a multitude of cells like endothelial cells, smooth muscle cells, and macrophages. These cells express PARs that upon thrombin‐mediated receptor activation elicit a myriad of responses in the vascular endothelium, including permeability changes, mobilization of adhesive molecules to the endothelial surface, and stimulation of cytokine production such as prostaglandins and platelet‐activating factor (Coughlin, 2000). Correspondingly, PAR‐1 expression increases in balloon‐injured rat carotid artery and human atherosclerotic tissue. Mice deficient in PAR‐1 activity show significant increase in vascular smooth muscle cell density in normal blood vessels compared to wild‐type mice, without affecting vascular smooth muscle proliferation. Moreover, since activated PAR‐1 has been shown to stimulate collagen synthesis in vascular smooth muscle cells, extracellular matrix formation may be compromised due to an altered injury response in PAR‐1‐deficient mice (Cheung et al., 1999).

Another study demonstrating intercellular signaling between bacteria and host cells identified the role of bacterial cysteine proteinases (from *Porphyromonas gingivalis*) called gingipains in the activation of human PAR‐1 and PAR‐4 receptors present on the surface of platelets (Lourbakos et al., 2001). In humans, this bacterium causes periodontitis and associated cardiovascular disease. The latter effect on cardiovascular function could be explained by activation of PARs by bacterial proteinases that lead to platelet aggregation (Figure 4c). Inhibition of gingipains with leupeptin blocked platelet aggregation, indicating a key role for bacterial proteases in platelet aggregation. Similarly, preincubation of platelets with inhibitors of platelet activation, PGI2 or forskolin, completely inhibits the aggregation induced by proteases, further indicating that bacterial proteases cause platelet aggregation in the host (Lourbakos et al., 2001).

### 3.9 Cellular senescence

Cellular senescence has been found to be a key determinant of tissue and organismal aging. Proteases can influence cell senescence by modulating intracellular signaling and the expression of senescence regulators. In this context, the Su(var)3–9, Enhancer‐of‐zeste, and Trithorax domain‐containing protein 8 (SET8) enzyme modulates the expression of p21, which is induced in senescent cells. Specifically, the ubiquitin protease USP17 removes ubiquitin tags from SET8 and stabilizes it, which leads to transcriptional repression of p21 and avoidance of cellular senescence (Fukuura et al., 2019). Conversely, the serine protease inhibitor serpinB2 promotes senescence through the stabilization of p21 (Hsieh et al., 2017), presumably by preventing protease‐mediated degradation of p21.

In addition to proteases that modulate directly or indirectly p21 levels, other proteases were found to regulate cell senescence by impacting other cellular processes. For example, mitochondrial dysfunction has been previously found to be a determinant of cell senescence: Perturbation of the function of mitochondrial proteases such as IMMP2L contributes to this process. Normally, IMMP2L processes and thus activates the mitochondrial metabolic enzyme glycerol‐3‐phosphate dehydrogenase (GPD2) and the cell death regulator apoptosis‐inducing factor (AIF). Impediment of such IMMP2L‐dependent proteolysis drives senescence by reprogramming the redox status of mitochondria, phospholipid metabolism, and associated signaling and by preventing cell death (Sica & Kroemer, 2018; Yuan et al., 2018).

In addition to regulating senescence, other proteases have been found to mark specifically senescent cells. For example, DPP4 (dipeptidyl peptidase 4) is selectively found on the plasma membrane of senescent cells but not of proliferating fibroblasts (Kim et al., 2017). In addition, DPP4 is also necessary to drive cell senescence because its inhibition impedes this cellular reprogramming via activation of a protective AMPK/SIRT1/NRF2 signaling axis (Chen et al., 2020). Membrane proteases (such as DPP4) that are specific or enriched on the plasma membrane of senescent cells may modulate extracellular proteolysis together with extracellular proteases that are secreted by senescent cells as part of the senescence‐associated secretory phenotype (SASP; Lopes‐Paciencia et al., 2019), which also includes high levels of inflammatory cytokines, immune modulators, and growth factors. SASP proteases included matrix metalloproteases (MMPs), serine proteases, and regulators of the plasminogen activation pathway. SASP proteases may proteolytically cleave transmembrane receptors, leading to the release of soluble versions that may act as decoy receptors (Coppe et al., 2010). Moreover, SASP proteases have been proposed to degrade or modify the activity of signaling factors and components of the extracellular matrix. For example, the matrix metalloproteases MMP‐1, MMP‐3, and MMP‐10 are part of the SASP and contribute to proteolytically cleaving and activating signaling components of the SASP, such as interleukins and CXCL/CCL family members (Coppe et al., 2010).

Importantly, MMPs that are secreted by senescent cells have been found to drive tumorigenesis. Specifically, it was found that the survival of tumor cells implanted in immunocompromised mice is enhanced by the co‐transplantation of human senescent fibroblasts (generated via telomere shortening or via stress‐induced premature senescence caused by bleomycin), compared to non‐senescent fibroblasts. The pro‐tumorigenic effects of senescent fibroblasts seem to stem from matrix metalloproteinases (MMP) produced by cancer‐associated fibroblasts because injection of the MMP inhibitor GM6001 abolished the size and proliferation differences of tumor xenografts in vivo (Liu & Hornsby, 2007).

Altogether, these studies indicate that MMPs are key SASP proteases that influence disease pathogenesis. However, it is also interesting to note that another study has found a distinct outcome of knockout of another matrix metalloprotease, MMP14. Specifically, *Mmp14* ^−/−^ mice have increased numbers of p16 and p21‐positive senescent cells, higher β‐galactosidase activity, and inflammation associated with SASP (Gutierrez‐Fernandez et al., 2015). These findings suggest that MMP14 is necessary to prevent cell senescence.

Altogether, proteases influence the cellular reprogramming that leads to cell senescence and its outcome by acting cell‐autonomously and non‐autonomously as part of the SASP.

## 4 ADDITIONAL ROLES OF PROTEASES IN THE EXTRACELLULAR SPACE

As explained above, proteases play important roles in the extracellular space where they contribute to intercellular signaling and to the systemic outcome of the SASP of senescent cells. In this paragraph, we will discuss the additional role of proteases in the extracellular space.

The autophagy/lysosome and ubiquitin–proteasome system are major pathways for maintaining proteostasis but their action is limited to the intracellular space. Proteases have been detected in all intracellular compartments as well in the extracellular space and therefore may maintain proteostasis in the extracellular environment by acting on proteins of the extracellular matrix, the blood plasma, the interstitial fluid, and the lymph and transcellular fluid (e.g., cerebrospinal fluid) where the autophagy/lysosome and ubiquitin–proteasome are not present. In such extracellular environments, proteases may modify substrates (such as secreted signaling factors) to modulate their functions in intercellular signaling or, perhaps, to remove misfolded and hence dysfunctional signaling factors and other components of the extracellular environment.

Extracellular matrix (ECM) homeostasis is regulated by several proteases largely consisting of matrix metalloproteinases (MMPs), as previously reviewed in detail (Kessenbrock et al., 2010; Page‐McCaw et al., 2007). In addition, because the extracellular matrix sequesters many secreted signaling factors (Yue, 2014), extracellular proteases can also influence intercellular signaling by remodeling the extracellular matrix and rendering such matrix‐sequestered signaling factors bioavailable. In addition, many signaling factors are secreted as precursors that become active only after regulated proteolytic processing by extracellular proteases. An example of the importance of such protease‐mediated activation comes from the newt, a model organism with remarkable regenerative capacity. In this system, it was found that serum clotting proteases, that is, thrombin and plasmin, proteolytically activate BMP4 and BMP7 and increase BMP4/7 signaling, which promotes cell‐cycle re‐entry of satellite stem cells during muscle regeneration in limb‐amputated newts. Cell‐cycle re‐entry is repressed by inhibition of serine protease activity in vivo, which in turn can be rescued by cleaved‐mimic BMPs (Wagner et al., 2017). Altogether, this study provides an example of how extracellular proteases can impact regeneration by regulating the activity of circulating signaling factors.

In addition to regulating extracellular signaling, extracellular proteases can also provide a defense against extracellular pathogens and such immune response typically weakens with aging. For example, studies in *Drosophila* found that a circulating serine protease, Persephone, facilitates inflammatory responses via the systemic activation of the Toll pathway, which acts as a danger‐sensing mechanism either in response to endogenous molecules (Ming et al., 2014) as well as in response to microbial infections (Issa et al., 2018).

In addition to extracellular proteases, immune responses are also governed by intracellular proteases. For example, thymus‐specific protease TSSP/PRSS16 is present in the endosomes of thymic cortical epithelial cells and is required for maturation of the CD4+ T lymphocytes. TSSP‐deficient mice develop spontaneous tumors upon aging, indicating that optimal levels and function of this protease are needed to avoid age‐associated immunodeficiency, which is one of the factors that increase cancer risk with aging (Brisson et al., 2015).

Whereas proteases that activate immune responses are generally beneficial for health span via preservation of immune function, there are also examples of how chronic activation of such responses can be deleterious. For example, the complement factors B and C2 are atypical serine proteases that can recognize and eliminate pathogens by activating the complement pathway. However, excessive or chronic complement activation may result in uncontrolled production of complement fragments that can deposit and cause tissue damage in inflammatory conditions like rheumatoid arthritis and in ischemia/reperfusion injury (Sim & Tsiftsoglou, 2004), as well as in dementias like Alzheimer's disease (Kolev et al., 2009, 2010; Morgan, 2018). Altogether, these studies indicate that extracellular proteases can preserve health span by resolving microbial infections; however, chronic complement activation can lead to tissue damage and fuel the progression of age‐related disease.

Complement factors have also emerged as important modulators of non‐immune functions. In particular, Adipsin (also known as complement factor D) is a circulating serine protease secreted by adipocytes that improves pancreatic β‐cell function. Reduced Adipsin levels are found in the circulation of type‐2 diabetes (T2D) patients whereas high Adipsin levels predict a low risk of developing T2D (Tafere et al., 2020). Indeed, Adipsin knockout mice are hyperglycemic and insulin secretion is compromised whereas systemic replenishment of Adipsin treats hyperglycemic mice by increasing insulin secretion. Mechanistically, Adipsin generates complement C3a peptide, which binds to C3aR receptor on pancreatic beta cells and enhances insulin secretion in response to glucose administration (Lo et al., 2014).

In addition to activating immune responses that fight microbial infections, extracellular proteases have been found to preserve homeostasis also by removing misfolded proteins from the extracellular space (Genereux et al., 2015; Genereux & Wiseman, 2015; Wyatt et al., 2013). For example, it was found that the extracellular protease plasmin is induced in Alzheimer's disease and it constitutes an adaptive response that cleaves Alzheimer's associated amyloid‐beta and reduces its toxicity (Tucker et al., 2000). Interestingly, the levels of some extracellular proteases can be upregulated by pathogens, suggesting that, like for proteases involved in immune functions, extracellular proteases involved in protein quality control are linked with the response to infections (Gallotta et al., 2020). Indeed, it was found that several proteases known for their roles in innate immunity can also function to maintain protein quality control in the extracellular space in *C*. *elegans* (Gallotta et al., 2020).

Altogether, these studies indicate that extracellular proteases contribute to aging by regulating intercellular signaling, by remodeling the extracellular space, by modulating regeneration and immune responses, and by preserving protein quality control in the extracellular space (Figure 4).

## 5 REGULATION OF PROTEIN QUALITY CONTROL BY PROTEASES IN THE CONTEXT OF AGE‐RELATED NEURODEGENERATION

**FIGURE 5:**
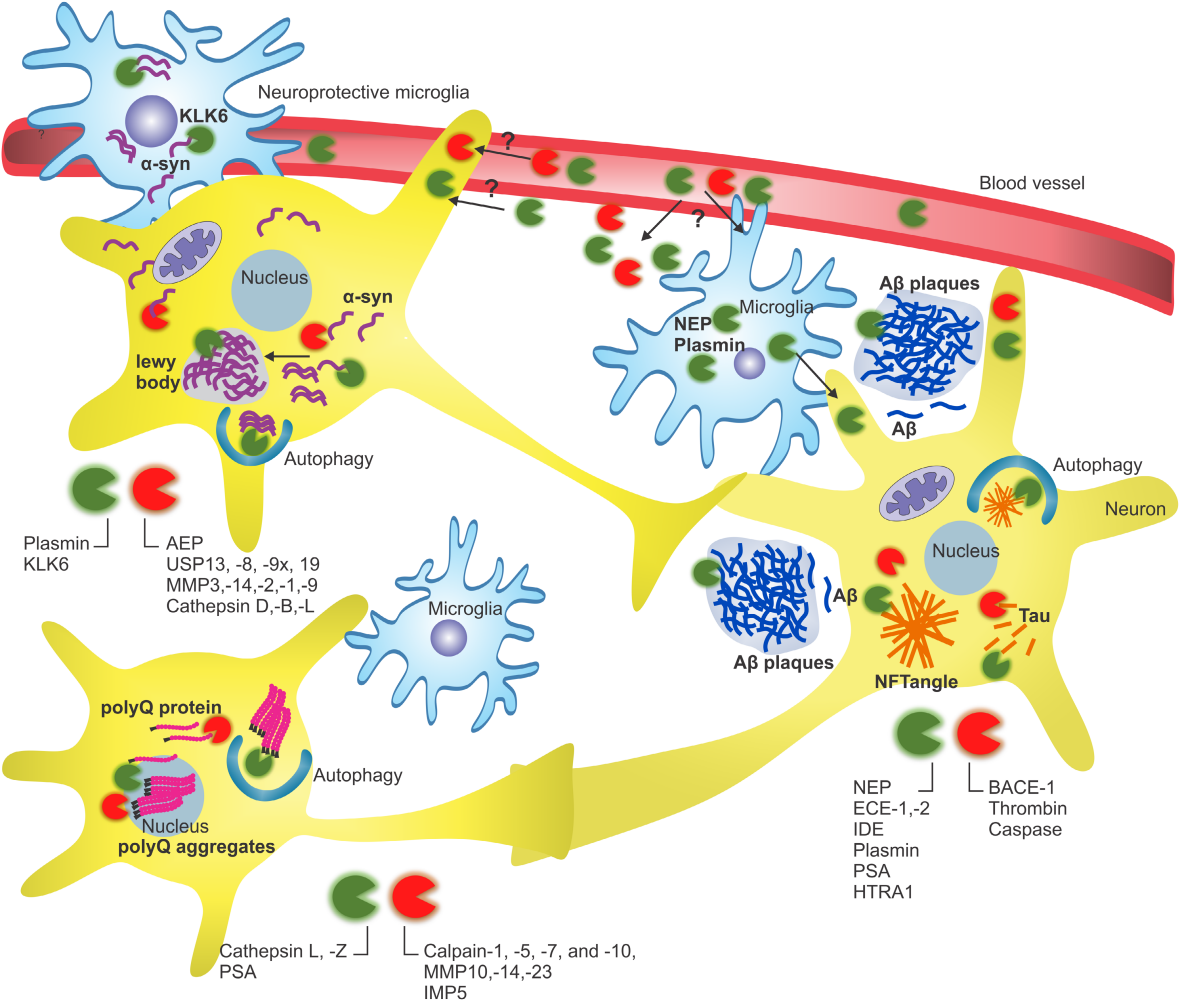
Role of proteases in neurodegeneration. Proteases can proteolytically cleave target proteins like APP, polyglutamine (polyQ), and tau into smaller toxic peptides that drive neurodegenerative diseases. On the contrary, other proteases digest toxic peptides either in the cytosol or in lysosomes and ameliorate neurodegenerative diseases

Loss of protein homeostasis during aging generally compromises tissue function and is also linked to the development of age‐related neurodegenerative diseases, which include polyglutamine (polyQ) disease, Alzheimer's disease (AD), Parkinson's disease (PD), amyotrophic lateral sclerosis (ALS), tauopathies, and Creutzfeldt–Jakob disease (CJD). In this section, we will discuss examples of proteases that can either aggravate or ameliorate neurodegeneration (Figure 5 and Table 2).

**TABLE 2:** Proteases that aggravate or improve neurodegenerative diseases

Neurodegenerative disease	Proteases	
	That aggravate the disease	That improve the disease
Polyglutamine diseases	Caspases, Calpain‐1, Calpain‐5, Calpain‐7, Calpain‐10, MMP‐10, MMP‐14, MMP‐23, MMP‐2, Signal peptide protease‐like, Intramembrane protease 5 (IMP5), SEC11 homolog A (SPC18)	Cathepsin L, Cathepsin Z, Puromycin‐sensitive aminopeptidase (PSA)
Parkinson's disease	Asparagine Endopeptidase (AEP), Cathepsin B, Cathepsin D, and Cathepsin L, Ubiquitin specific protease 13 (USP13), MMP‐3, MMP‐14, MMP‐2, MMP‐1, MMP‐9, Calpains	Calpains, Plasmin, Neurosin (kallikrein‐6/KLK6)
Alzheimer's disease	β‐secretase (β‐site APP cleaving enzyme 1 or BACE1), Thrombin and thrombin‐like proteases, Caspases, Asparagine Endopeptidase (AEP)	Neprilysin (NEP), Endothelin Converting Enzymes 1 and 2 (ECE‐1, ECE‐2), MMPs (CD147, MMP‐2, MMP‐9), ADAM10, Plasmin, Puromycin‐sensitive aminopeptidase (PSA), High Temperature Requirement Serine Protease A1 (HTRA1)

## 6 POLYGLUTAMINE DISEASES

### 6.1 Proteases that aggravate polyglutamine diseases

Expansion of polyglutamine (polyQ) repeats in specific proteins leads to neurodegenerative diseases such as Huntington's disease (HD) and spinocerebral ataxia (SCA1‐7) due to the proteotoxicity of these aggregation‐prone proteins (Bates et al., 2015; Chuang & Demontis, 2021; Labbadia & Morimoto, 2013). Proteolytic cleavage of huntingtin (Htt) is a key event in HD progression and truncated huntingtin fragments generally yield greater cellular toxicity than full‐length Htt. Caspases and calpains are widely studied proteases in the pathophysiology of HD. There are two sites in Htt that calpains can cleave, which leads to the production of N‐terminal fragments. Expression of calpain‐resistant mutant Htt in 293T cells reduces Htt proteolysis and aggregation (Gafni et al., 2004). Additionally, calpain family members (calpain‐1, calpain‐5, calpain‐7, and calpain‐10) increase in expression or activity alongside Htt fragmentation in cell and mouse models of HD, suggesting that they may contribute to Htt proteolysis and disease pathology (Gafni et al., 2004).

An unbiased screen of 514 siRNAs targeting proteases led to the identification of novel proteases that regulate Htt toxicity, including matrix metalloproteinases (MMPs). Specifically, MMP activation increases in striatal cells expressing Htt‐polyQ, and inhibition of MMP‐10, MMP‐14, and MMP‐23 reduces toxicity (Miller et al., 2010). Similarly, inhibition of *Drosophila* MMP2 results in robust improvements of motor function in HD model flies (Miller et al., 2010). Moreover, loss of other proteases also ameliorates Htt toxicity: these include signal peptide protease‐like, an amino‐terminal signal peptide protease, intramembrane protease 5 (IMP5), SEC11 homolog A (SPC18), and calpain‐5 and calpain‐7 (Miller et al., 2010). However, considering that MMPs are extracellular proteases, it remains undetermined how they can act on Htt‐polyQ proteins, which are intracellular. Possibly, MMPs proteolytically cleave Htt‐polyQ that is secreted or released upon cell death.

### 6.2 Proteases that improve polyglutamine diseases

The three types of active sites found in eukaryotic proteasomes cleave very poorly—if at all—within polyQ sequences, which are often released intact from the proteasome. Puromycin‐sensitive aminopeptidase (PSA) is the only cytosolic protease capable of digesting polyQ sequences (Bhutani et al., 2007). Consequently, PSA overexpression reduces toxicity and prevents the accumulation of mutant huntingtin aggregates in culture and in vivo. However, because PSA is an exopeptidase, its capacity to degrade long polyQ stretches via direct cleavage is inefficient.

Interestingly, PSA can also clear mutant huntingtin via its capacity to induce/increase the rate of autophagy, although the underlying mechanisms remain to be elucidated (Menzies et al., 2010). In agreement with the role of autophagy in the clearance of aggregation‐prone proteins, lysosomal extracts are found to digest polyQ sequences at a rate 100‐fold greater than cytosolic extracts. Cathepsins L and Z are the lysosomal proteases responsible for polyQ digestion. Given that cathepsin L is an endopeptidase, it likely carries out the initial cleavage events, generating shorter peptides that are then cleaved down to amino acids by the carboxypeptidase cathepsin Z (Bhutani et al., 2012).

## 7 PARKINSON’S DISEASE

### 7.1 Proteases that aggravate Parkinson's disease

Parkinson's disease (PD) is characterized by abnormal α‐synuclein (α‐syn) aggregates, referred to as Lewy bodies, which are linked with dopaminergic neurodegeneration (Bloem et al., 2021; Poewe et al., 2017). Cleavage of α‐syn by asparagine endopeptidase (AEP), an endolysosomal cysteine protease, generates an α‐syn fragment (α‐syn N103) that aggregates much faster than full‐length α‐syn. The resulting α‐syn N103 fibrils recruit full length α‐syn into aggregates and cause higher neurotoxicity and degeneration of dopaminergic neurons (Kang et al., 2018). Consequently, mice with AEP overexpression exhibit motor function impairment and dopaminergic neurodegeneration (Kang et al., 2018). Likewise, α‐syn N103 is more abundant in the brains of PD patients than controls. Conversely, the expression of a non‐cleavable α‐syn mutant partially rescues these phenotypes (Zhang et al., 2017). Furthermore, AEP activity may play a role also in drug‐induced PD resulting from MPTP (N‐methyl‐4‐phenyl‐1,2,3,6‐tetrahydropyridine) exposure.

The lysosomal proteases cathepsin B, D, and L have also been shown to generate C‐terminally truncated α‐syn fragments: >60% of α‐syn cleavage sites can indeed be assigned to lysosomal proteases. Purified brain lysosomes from control and transgenic PD mouse models show enrichment of cysteine cathepsins without a change in individual cathepsin activities (McGlinchey et al., 2019). It was also suggested that incomplete digestion of aggregated α‐syn by lysosomes may seed amyloid formation (McGlinchey et al., 2019). Despite a similar contribution to the pathogenesis of PD, AEPs and cathepsins seems to cleave α‐syn at distinct sites (McGlinchey et al., 2019). It was also reported that AEP can leak from lysosomes into the cytosol where it can promote α‐syn aggregates (Zhang et al., 2017).

Another protease, ubiquitin‐specific protease 13 (USP13), which functions as a deubiquitinase (DUB), significantly increases in the midbrain of PD patients. USP13 knockdown increases ubiquitination and clearance of α‐syn and promotes survival of dopaminergic neurons (Liu et al., 2019). Similarly, knockdown of USP8, USP9x, and USP19 reduces α‐syn toxicity (Stefanis et al., 2019). Therefore, increased DUB expression in PD may interfere with the proteasomal‐mediated degradation of α‐syn by decreasing its ubiquitination. Moreover, it is also possible that modulation of DUBs in PD alters the levels of signaling factors that regulate the capacity of the cell to cope with α‐syn‐induced toxicity.

Metalloproteinases such as MMP3, MMP14, MMP2, MMP1, and MMP9 (in order of efficiency) cleave α‐syn in vitro (Sung et al., 2005). MMP3 cleaves α‐syn from its C‐terminal end and generates α‐syn fragments that aggregate more readily and are more toxic to cells than non‐cleaved α‐syn. Interestingly, α‐syn overexpression results in increased MMP3 expression both in cells and in a rat model of PD (Sung et al., 2005). However, as explained above for polyglutamine diseases, it is unclear how extracellular MMPs may target α‐syn, which is an intracellular pre‐synaptic protein. Possibly, MMPs act on extracellular α‐syn that is secreted or released upon cell death, and hence, MMPs may regulate the cell‐to‐cell propagation of these pathogenic proteins across the brain (Lee et al., 2014; Yamada & Iwatsubo, 2018).

Beyond α‐synuclein, other proteins relevant for PD and dopaminergic function are targets of proteases, including tyrosine hydroxylase (TH). TH catalyzes the rate limiting step in dopamine synthesis and is a substrate of calpains (Kiuchi et al., 1991), Ca^2+^‐sensitive proteases that are chronically activated in PD and other neurodegenerative diseases because of defective calcium homeostasis (Vosler et al., 2008). Normally, α‐syn reduces TH activity and dopamine biosynthesis (Tehranian et al., 2006) but sequestration of α‐syn into aggregates renders TH available for proteolysis by calpains. Calpain‐mediated proteolysis at the N‐terminus of TH increases its activity, and this leads to the production of toxic levels of dopamine (Kiuchi et al., 1991; Vosler et al., 2008). Examination of human PD midbrain samples indeed indicates higher calpain activity in dopaminergic neurons compared to controls (Crocker et al., 2003). Likewise, increased calpain‐mediated proteolysis is seen in nigral dopamine neurons in MPTP‐induced PD: inhibition of calpain activity, either by use of an inhibitor (MDL‐28170) or by adenovirus‐mediated overexpression of calpastatin, significantly reduces loss of dopaminergic neurons and restores motor functionality in MPTP‐treated mice (Crocker et al., 2003).

Altogether, these studies suggest that calpain activation in PD deranges the homeostatic regulation of dopamine biosynthesis via proteolysis‐mediated activation of TH. Although this may initially constitute an adaptive stress response to potentiate dopaminergic functions, the higher levels of dopamine are toxic, especially for cells that express mutant α‐syn (Tabrizi et al., 2000), and they may thus further drive the degeneration of dopaminergic neurons in PD.

### 7.2 Proteases that improve Parkinson's disease

Parkin is an E3 ubiquitin ligase that is involved in target protein ubiquitination and mitophagy. Mutations in parkin cause early‐onset autosomal recessive juvenile Parkinsonism (AR‐JP) due to the accumulation of abnormal (dysfunctional) mitochondria in the brain. Simultaneous overexpression of parkin and α‐syn ameliorates α‐syn‐related cell death in a conditionally immortalized embryonic hippocampal cell line (H19‐7). Parkin increases the rate of α‐syn degradation via activation of calpain, and this effect is abrogated by calpeptin/calpastatin, a calpain inhibitor (Kim et al., 2003). Calpain truncates α‐syn at its N‐terminus, which removes the non‐amyloid component and likely inhibits the organization of α‐syn into fibrils, thereby facilitating its clearance. Therefore, different from studies on MPTP‐induced PD (see above), calpain seems to be neuroprotective in AR‐JP (Kim et al., 2003).

Different from calpains, which are intracellular proteases, other proteases located in the extracellular space may target the extracellular pool of α‐syn and prevent its cell‐to‐cell propagation across the brain. These include plasmin, which cleaves α‐syn at its N‐terminus, mainly within the KTKEGV repeat regions. Interestingly, α‐syn variants with point mutations (A30P, E46K, and A53T) seen in patients with early‐onset familial PD are cleaved by plasmin with equal efficiency compared to WT α‐syn (Kim et al., 2012), and, as explained above, plasmin physiological function may primarily consist in the proteolysis and inhibition of cell‐to‐cell propagation of extracellular α‐syn (Kim et al., 2012).

Like calpains and plasmin, neurosin (kallikrein‐6/KLK6) is a serine protease capable of cleaving α‐syn. Down‐regulation of KLK6 is associated with increased accumulation of α‐syn in PD patients and mouse PD models. Mature neurosin autocatalytically inactivates itself via cleavage at R80, which leads to its short half‐life. A brain‐targeted, stabilized KLK6 variant with a longer half‐life (R80Q) degrades α‐syn in oligodendrocytes and astrocytes, ameliorates myelin sheath deficiency in the corpus callosum and improves behavioral deficits in a PD mouse model obtained via α‐syn overexpression (Spencer et al., 2015). Like plasmin, KLK6 primarily degrades extracellular rather than intracellular α‐syn and avoids its intercellular propagation. Additionally, α‐syn cleaved by KLK6 is taken up by microglial cells but the wild‐type full‐length α‐syn is not, indicating that KLK6‐mediated cleavage may facilitate the clearance of α‐syn by the microglia (Spencer et al., 2015). Because KLK6 levels are reduced in patients with synucleinopathies including PD (Pampalakis et al., 2017), restoring KLK6 levels may represent a possible therapeutic intervention for these neurodegenerative diseases.

## 8 ALZHEIMER’S DISEASE

### 8.1 Proteases that aggravate Alzheimer's disease

Alzheimer's disease (AD) is a complex neurodegenerative disease which is characterized by the formation of extracellular amyloid beta (Aβ) plaques and intracellular tau/MAPT aggregates in the brain (Masters et al., 2015; Scheltens et al., 2021).

Sequential cleavage by β‐ and γ‐secretases mediates the conversion of a type I transmembrane protein called β‐amyloid precursor protein (βAPP) into Aβ peptides. Multimeric γ secretase acts in a complex with presenilin (PS1 or PS2), nicastrin, APH1, and PEN‐2. Mutations in these genes cause the majority of familial cases of AD (Mucke, 2009). β‐secretase (β‐site APP cleaving enzyme 1 or BACE1) can cleave Aβ1‐40/1‐42 at the C‐terminus to generate shorter Aβ peptides (Aβ1‐34). BACE1‐deficient mice have high neonatal mortality, smaller size, and hyperactive behavior. BACE2, a paralog of BACE1, is expressed in glial cells and may contribute to Aβ production in these cells (Dominguez et al., 2005). However, unlike BACE1, BACE2 was identified as a protease with strong capacity to cleave Aβ, though BACE2 degrades only Aβ monomers but not fibrils (Abdul‐Hay et al., 2012).

In addition to proteases that generate Aβ peptides, other proteases have been found to regulate AD. Thrombin and thrombin‐like proteases are elevated in AD whereas the protease nexin that inhibits thrombin is depleted in the brains of AD patients (Wagner et al., 1989). Thrombin is a trypsin‐like serine protease of the blood coagulation system and it is generated by proteolytic cleavage of its precursor, prothrombin, which is expressed in the brain but is primarily made by the liver and released into the bloodstream (Ben Shimon et al., 2015). Thrombin can cleave pathogenic tau at five different sites and is present in tau neurofibrillary tangles in AD brains. Phosphorylation of tau by glycogen synthase kinase‐3β (GSK‐3β) inhibits proteolytic processing by thrombin, apart from cleavage at R155‐G156. In addition, the molecular weight of the N‐terminal tau fragment released by thrombin cleavage is similar to that of the N‐terminal tau fragment found in the cerebrospinal fluid of AD patients (Arai et al., 2005).

A study in N2a neuroblastoma cells overexpressing an aggregation‐prone tau with a mutation in the microtubule‐binding repeat domain (K18ΔK280) indicates that tau fragmentation precedes its aggregation (Khlistunova et al., 2006). Fragmentation of the tau repeat domain is decreased when tau is phosphorylated. Because the major cleavage occurs at K257‐S258, which is a major proteolytic site for thrombin, this finding suggests that thrombin cleaves tau. Although fragment 1 (F1) generated from the initial thrombin‐mediated cleavage is mostly soluble, subsequent C‐terminal digestions (F2 and F3) render the fragment susceptible to aggregation (Khlistunova et al., 2006). Because the generation of F1 tau fragment is a prerequisite for subsequent cleavage events that generate aggregation‐prone F2 and F3 fragments, inhibition of the initial thrombin‐like cleavage may stop production of F2/F3, which can aggregate and even nucleate the aggregation of full‐length tau (Wang et al., 2007).

In addition to thrombin, caspases can cleave tau at D421 and this event accelerates tau fibrillization by nucleating aggregate formation (Gamblin et al., 2003). Injection of a viral vector for expression of caspase‐truncated tau into brains of wild‐type mice results in neurofibrillary tangle formation. Endogenous tau colocalizes with these neurofibrillary tangles, suggesting that caspase‐truncated tau can nucleate the aggregation of full‐length tau (de Calignon et al., 2010). Similar to AEP‐generated α‐syn fragments (see section 5.3), AEP‐mediated cleavage of tau results in fragments that are more prone to fibrillization. AEP also mediates cleavage of APP at N373 and N585, which results in β amyloid generation (Zhang et al., 2017). Altogether, multiple proteolytic events mediated by several proteases can initiate and promote the pathogenesis of AD and related conditions.

### 8.2 Proteases that improve Alzheimer's disease

Neprilysin (NEP) is a membrane‐bound zinc metalloprotease that degrades monomeric Aβ but does not degrade aggregates/oligomers. It acts as an ectoenzyme with the active site facing the extracellular side of the membrane (Betts et al., 2008). Levels of Neprilysin are often reduced with aging and in AD patient brains. Overexpression of Neprilysin in the skeletal muscle of transgenic AD mice reduces Aβ peptides as well as deposits in the brain without any side effects. The mechanism appears to consist in the degradation of circulating Aβ that comes in contact with muscle (Liu et al., 2009). Apart from direct digestion of Aβ, NEP can also proteolytically process neuropeptide Y into C Terminal Fragments that exert a neuroprotective effect presumably via activation of Y2 receptors in the hippocampus (Rose et al., 2009).

NEP2 also degrades Aβ peptides efficiently. NEP2 knockout increases Aβ levels with the most prominent effects observed in the hippocampus and brainstem/diencephalon. Treatment with phosphoramidon/thiorphan (inhibitors of M13 metalloproteases including NEP) produces a ~30‐fold to 50‐fold increase in Aβ accumulation. By contrast, NEP/NEP2 double‐knockout mice exhibit only modest increases (1.5–2.0‐fold) in Aβ, indicating that additional proteases sensitive to these inhibitors are likely involved in Aβ catabolism (Hafez et al., 2011). Such proteases may include endothelin converting enzymes 1 and 2 (ECE‐1 and ECE‐2), which are M13 family metalloproteases that can also be inhibited by phosphoramidon/thiorphan. Mice lacking ECE‐1 and ECE‐2 display a significant increase in Aβ peptides in the brain compared to age‐matched controls (Eckman et al., 2003). Transfection of cells that lack endogenous ECE activity with either the plasma membrane‐localized ECE‐1a isoform or with the intracellular ECE‐1b variant reduces Aβ peptide accumulation in culture media (Eckman et al., 2001), presumably because of intracellular Aβ proteolysis and consequently reduced secretion (Eckman et al., 2001, 2003).

As for other pathogenic proteins involved in neurodegeneration (see Sections 5.1 and 5.3), MMPs have been reported to modulate also Aβ degradation. Specifically, CD147 (also known as extracellular matrix metalloproteinase inducer/EMMPRIN) is a multifunctional cell surface type I transmembrane protein that stimulates the secretion of MMPs. On this basis, CD147 influences extracellular Aβ degradation indirectly by modulating MMP levels (Vetrivel et al., 2008). In particular, MMP‐2 and MMP‐9 are produced by astrocytes and detected near Aβ plaques and their expression increases in the cerebral cortex and hippocampus of aged APP/PS1 mice compared to age‐matched controls. Incubation of Aβ with astrocyte conditioned media (ACM) results in a decline in Aβ levels and generation of Aβ fragments characteristic of MMP‐mediated proteolysis. Aβ degradation in media conditioned with astrocytes from MMP‐2‐ or MMP‐9‐deficient mice is compromised compared with corresponding controls (Yin et al., 2006), indicating that astrocyte‐derived MMP‐2 and MMP‐9 indeed contribute to Aβ degradation.

A number of other proteases have been reported to modulate AD pathogenesis. These include tissue‐type plasminogen activator (tPA), which is activated upon binding to fibrin/Aβ aggregates and converts plasminogen to plasmin, an extracellular protease (see Section 5.4) that degrades monomeric and aggregated Aβ (Tucker et al., 2000a, 2000b). tPA is upregulated in the brains of AD mice but plasmin activity is low because plasminogen activator inhibitor 1 (PAI‐1) binds irreversibly to tPA to inhibit its protease activity. PAI‐1 is highly expressed in the vicinity of Aβ deposits and sites of inflammation in the brain of AD patients, aged mice, and transgenic APP mice (Jacobsen et al., 2008). Inhibition of PAI‐1 in AD mice (Tg2576) reduces Aβ levels in the brain and nearly completely ameliorates learning and memory (Jacobsen et al., 2008). Whereas tPA needs to bind fibrin/Aβ aggregates to be maximally active, urokinase‐type PA (uPA) is constitutively active (Tucker et al., 2002): both uPA and tPA clear Aβ aggregates via their capacity to promote the conversion of plasminogen to plasmin (Tucker et al., 2000, 2002).

Altogether, the examples above highlight how interventions that boost the levels and activity of extracellular proteases ameliorate AD pathogenesis at least in part by targeting Aβ aggregates. Likewise, there are several examples of intracellular proteases that target pathogenic tau/MAPT. These include puromycin‐sensitive aminopeptidase (PSA) (see Section 5.2).

In frontal temporal dementia (FTD), AD, and mouse tauopathy models, the cerebellum is one of the brain regions least affected by neurodegeneration. Interestingly, in postmortem human samples, PSA expression is higher in the cerebellum than cortex in both FTD and control brains. Overexpression of PSA in *Drosophila* tauopathy models results in amelioration of the “rough eye” phenotype (indicative of neurodegeneration) induced by both WT and P301L tau, and reductions in tau immunoreactivity (Karsten et al., 2006). hPSA/TAU^P301L^ double overexpressing transgenic mice exhibit delayed manifestation of paralysis, improved motor neuron density in the spinal cord, and rescue of the gliosis and axonal degeneration observed in mice overexpressing the tau mutant protein alone (Kudo et al., 2011). Additionally, these double overexpressing transgenic mice show significantly less tau in their cortex, cerebellum, brain stem, and spinal cord than controls for all age groups tested (Kudo et al., 2011).

Similar to its capacity to protect against tau, PSA overexpression reduces Aβ levels and toxicity in *Drosophila*, leading to restoration of normal eye development and increased median lifespan and locomotor function (Kruppa et al., 2013). However, the mechanism via which this occurs is unknown as PSA does not degrade Aβ in vitro. Additionally, both catalytically inactive and wild‐type PSA rescue Aβ toxicity in SH‐SY5Y cells. This finding rules out PSA‐mediated autophagy induction as a mechanism for Aβ clearance because the aminopeptidase function of PSA is required for autophagy induction by PSA (Kruppa et al., 2013).

Lastly, human high temperature requirement serine protease A1 (HTRA1) also degrades Aβ peptides, full‐length tau, tau fragments, as well as aggregated tau (both amorphous aggregates and ordered, paired helical filaments). Interestingly, HTRA1 mRNA levels increase in cells overexpressing pathogenic tau, and HTRA1 colocalizes with tau: this suggests that HTRA1 may be part of an adaptive stress response induced by tau. HTRA1 is also likely to have chaperone properties due to the propensity of its PDZ domain to bind to misfolded protein substrates (Clausen et al., 2011; Tennstaedt et al., 2012). On this basis, HTRA1 may have the broad capacity to target a variety of misfolded and aggregation‐prone proteins during normal brain aging and hence may impede age‐related neurodegeneration by preserving proteostasis.

## 9 CONCLUSIONS AND FUTURE DIRECTIONS

In this review, we have highlighted how proteases are a diverse family of proteolytic enzymes located in all cell compartments, including the cytosol, organelles, transmembrane/intramembrane spaces, and the extracellular space. Proteases can also act systemically to regulate cellular processes in tissues distant from their original source. By virtue of their proteolytic activity, proteases regulate the levels and activity of signaling factors and degrade misfolded and pathogenic proteins in all cell compartments. Owing to such diverse areas of action, proteases regulate all hallmarks of aging and regulate many biological processes that are deranged in age‐related diseases. In the context of age‐related neurodegeneration, some proteases have been found to contribute to disease initiation and progression by generating toxic and aggregation‐prone protein fragments. However, other proteases can degrade pathogenic proteins and hence reduce the progression and severity of neurodegeneration. Therefore, these findings indicate that targeted modulation of protease activity may provide therapeutic options for resolving age‐related dementias.

Throughout the review, we have outlined areas of innovative research on proteases. Given the vastity of the protease superfamily (~600), much remains to learn about the modulation of individual proteases during aging and in age‐related diseases. Questions that remain largely unanswered include (1) the mechanisms that control the proteolytic activity of proteases and impede their unregulated and indiscriminate action on target and non‐target proteins; (2) how misfolded and pathogenic proteins can be specifically targeted to protease‐mediated degradation; (3) how proteases synergize with other proteolytic systems (ubiquitin–proteasome and autophagy‐lysosome systems); and (4) how the activity of proteases in the extracellular space provides a unique means for controlling proteostasis in the circulation and in other extracellular environments that lack the proteostasis‐ensuring mechanisms conferred by intracellular proteolytic systems (ubiquitin–proteasome and autophagy‐lysosome).

In addition to addressing fundamental questions about the mechanisms of action and regulation of proteases during aging and age‐related diseases, future studies should aim at identifying suitable interventions to improve health span and delay the progression of age‐related disease by targeting proteases. These may consist of gene therapy and pharmacological approaches to modulate the activity of disease‐relevant proteases. Moreover, proteases could be re‐purposed to degrade disease‐causing proteins even if that is not their physiological function, in a manner similar to how the ubiquitin–proteasome system is hijacked for achieving targeted degradation of pathogenic proteins by using PROTACs (Cromm & Crews, 2017; Konstantinidou et al., 2019; Lai & Crews, 2017; Paiva & Crews, 2019; Pettersson & Crews, 2019). On this basis, a PROTAC‐like system could be designed to promote the specific proteolysis of disease‐causing proteins by proteases rather than by the proteasome. Such protease‐based PROTAC‐like system would expand the repertoire of interventions available to target disease‐causing proteins and may also reach pathogenic proteins located in compartments such as the extracellular space that are otherwise not amenable to proteasome‐mediated degradation. Moreover, contrary to classical PROTAC, which leads to proteasome‐mediated target protein degradation, such protease‐based PROTAC would provide a versatile system for controlled proteolysis that, depending on the protease and substrate, leads to complete target protein degradation or, conversely, to a modulatory cleavage that increases or refines the activity of target proteins.

Altogether, a better understanding of the function of proteases in aging and age‐related disease would provide a more comprehensive understanding of the mechanisms that control proteostasis during aging, and also offer the knowledge base for potential new therapeutic interventions for age‐related diseases.

## CONFLICT OF INTERESTS

The authors declare no conflicts of interest.

## AUTHOR CONTRIBUTIONS

MR, MC, and FD produced the initial draft of the review, with help from ZC. MR, MC, ZC, and FD reviewed the article, contributed to the content, and agreed on the final version. MR and MC contributed equally as first authors.

## Data Availability

Not applicable.
